# TorchMD: A Deep Learning Framework for Molecular Simulations

**DOI:** 10.1021/acs.jctc.0c01343

**Published:** 2021-03-17

**Authors:** Stefan Doerr, Maciej Majewski, Adrià Pérez, Andreas Krämer, Cecilia Clementi, Frank Noe, Toni Giorgino, Gianni De Fabritiis

**Affiliations:** † Acellera, 08005 Barcelona, Spain; ‡ Computational Science Laboratory, Universitat Pompeu Fabra, 08003 Barcelona, Spain; ¶ Department of Mathematics and Computer Science, Freie Universität, 14195 Berlin, Germany; § Department of Physics, Freie Universität, 14195 Berlin, Germany; ∥ Department of Chemistry, Rice University, Houston, 77005 Texas, United States; ⊥ Biophysics Institute, National Research Council (CNR-IBF), 20133 Milano, Italy; # Department of Biosciences, Università degli Studi di Milano, 20133 Milano, Italy; ∇ Institució Catalana de Recerca i Estudis Avançats, 08010 Barcelona, Spain

## Abstract

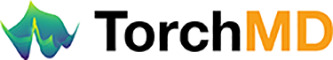

Molecular dynamics simulations provide a mechanistic description of molecules by relying on empirical potentials. The quality and transferability of such potentials can be improved leveraging data-driven models derived with machine learning approaches. Here, we present TorchMD, a framework for molecular simulations with mixed classical and machine learning potentials. All force computations including bond, angle, dihedral, Lennard-Jones, and Coulomb interactions are expressed as PyTorch arrays and operations. Moreover, TorchMD enables learning and simulating neural network potentials. We validate it using standard Amber all-atom simulations, learning an ab initio potential, performing an end-to-end training, and finally learning and simulating a coarse-grained model for protein folding. We believe that TorchMD provides a useful tool set to support molecular simulations of machine learning potentials. Code and data are freely available at github.com/torchmd.

## 1 Introduction

Classical molecular dynamics (MD) is a compute-intensive technique that enables quantitative studies of molecular processes. Of the possible modeling approaches, classical all-atom MD represents all of the atoms of a chosen system explicitly (including solvent) and accounts for interatomic forces through classical bonded and nonbonded potentials. It has seen remarkable developments due to its fidelity, and it has been applied with success to problems such as conformational changes, folding, binding, permeation, and many others.^1^ It has, however, faced two significant challenges: first, the calculation of the tables of interatomic potentials known as force fields^2^ has traditionally been highly time-consuming and requires significant fine-tuning; second, it is compute-intensive, and despite heroic efforts and progress in accelerating MD codes,^3^ it still struggles to reach the temporal scales of several important physiological processes.

Machine learning (ML) potentials have become especially attractive with the advent of deep neural network (DNN) architectures, which enable the example-driven definition of arbitrarily complex functions and their derivatives. As such, DNNs offer a very promising avenue to embed fast-yet-accurate potential energy functions in MD simulations, after training on large-scale databases obtained from more expensive approaches. One particularly interesting feature of neural network potentials is that they can learn many-body interactions. The SchNet architecture,^4,5^ for instance, learns a set of features using continuous filter convolutions on a graph neural network and predicts the forces and energy of the system. SchNet was originally used in quantum chemistry to predict energies of small molecules from their atomistic representations. A key feature of using SchNet is that the model is inherently transferable across molecular systems. More recently, this has been extended to learn a potential of mean force which involves averaging of a potential over some coarse-grained degrees of freedom,^6−12^ which however pose challenges in their parametrization.^13,14^ Indeed, molecular modeling on a more granular scale has been tackled by so-called coarse-graining (CG) approaches before,^15−20^ but it is particularly interesting in combination with DNNs.

Here, we introduce TorchMD, a molecular dynamics code built from scratch to leverage the primitives of the ML library PyTorch.^21^ TorchMD enables the rapid prototyping and integration of machine-learned potentials by extending the bonded and nonbonded force terms commonly used in MD with DNN-based ones of arbitrary complexity. The two key points of TorchMD are that, being written in PyTorch, it is very easy to integrate other ML PyTorch models, like ab initio neural network potentials (NNPs)^5,22^ and machine learning coarse-grained potentials.^8,9^ Second, TorchMD provides the capability to perform end-to-end differentiable simulations,^14,23,24^ being differentiable on all of its parameters. Similarly, Jax^25^ was used to perform end-to-end differentiable molecular simulations on Lennard-Jones systems^26^ and for biomolecular systems as well.^27^ Other efforts have tackled the integration of MD codes with DNN libraries, although in different contexts. For all-atom models, Wang et al.^23^ demonstrated the use of graph networks to recover empirical atom types. Ab initio QM-based training of potentials is being tackled by several groups, including Gao et al.,^22^ Yao et al.,^28^ and Schütt et al.^29^ but not using a differentiable PyTorch environment.

This paper provides an account of the capabilities of TorchMD (Section 2), highlighting the functional forms supported and an effective fitting strategy for data-driven DNN potentials. All of the TorchMD code, including a tutorial on coarse-graining the chignolin protein and the corresponding training data, is open-source and available at github.com/torchmd.

## 2 Methods

### 2.1 TorchMD Simulations

TorchMD is, at first glance, a standard molecular dynamics code. It offers NVT ensemble simulations including a Langevin thermostat. Starting atomic velocities are derived from a Maxwell–Boltzmann distribution. Integration is done using the velocity Verlet algorithm. Long-range electrostatics are approximated using the reaction field method.^30^ TorchMD also supports simulations of periodic systems. Minimization is done using the L-BFGS algorithm. Because it is written in Python using PyTorch arrays, it is also very simple to modify, and simulations can be run on any devices supported by PyTorch (CPU, GPU, TPU). However, unlike specialized MD codes^31^ it is not designed for speed. TorchMD uses chemical units consistent with classical MD codes such as ACEMD,^31^ namely kcal/mol for energies, K for temperatures, g/mol for masses, and Å for distances.

### 2.2 Analytical Potentials

**Figure 1:**
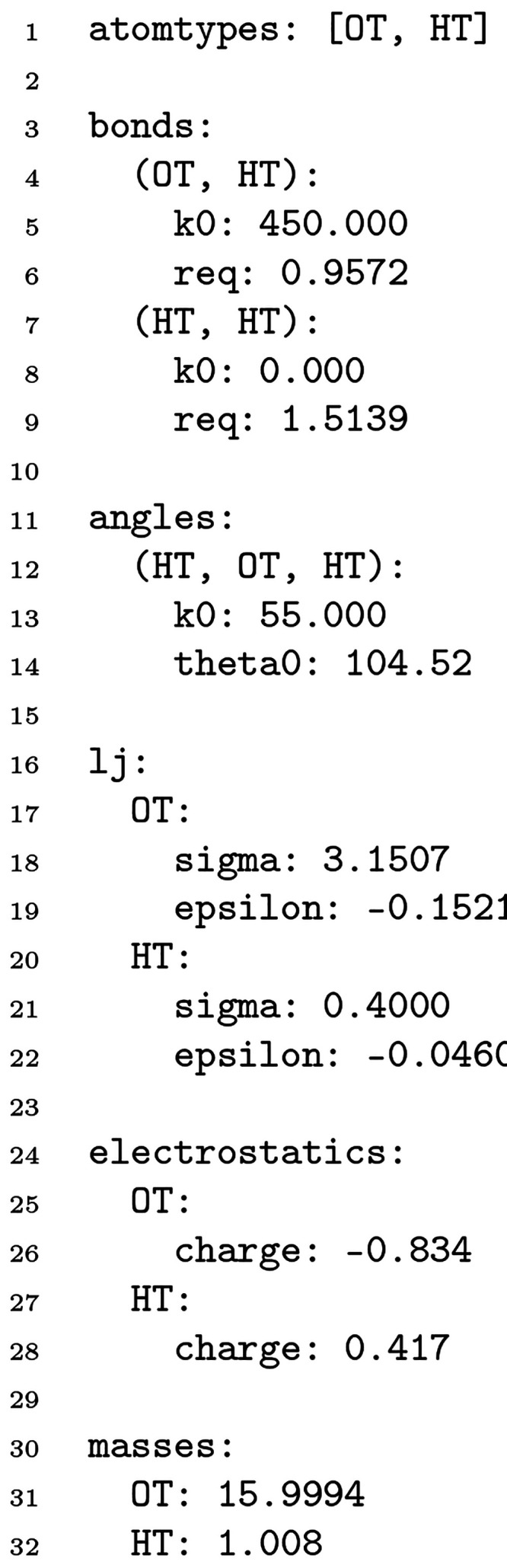
An example YAML force field for water molecules.

TorchMD supports reading AMBER force-field parameters through parmed.^32^ In addition to that, to allow for faster prototyping and development, it implements its own easy to read YAML-based force-field format. An example YAML force-field file for the simulation of a water box is given in Figure 1. Currently, TorchMD’s missing features include hydrogen bond constraints and neighbor lists.

TorchMD implements the functional form of the AMBER potential.^33^ It offers all basic AMBER terms: harmonic bonds, angles, torsions, and nonbonded van der Waals and electrostatic energies. The above potentials are implemented as follows. The bonded potential terms are calculated as
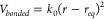
where *k*_0_ is the force constant, *r* is the distance between the bonded atoms, and *r*_*eq*_ is the equilibrium distance between them.

The angle terms are calculated as
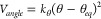
where θ is the angle between the three bonded atoms, *k*_θ_ is the angular force constant, and θ_*eq*_ is the equilibrium angle.

The torsion terms are calculated as
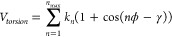
where ϕ is the dihedral angle between the four atoms, γ is the phase offset, and *k*_*n*_ is the amplitude of the harmonic component of periodicity *n*.

The nonbonded van der Waals (VdW) terms are calculated as
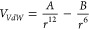
where *A* = 4*ϵσ*^12^ and *B* = 4*ϵσ*^6^ with ϵ being the well depth of the interaction of two atoms, and σ is the distance at which the energy is zero. The VdW potential also supports a cutoff by using a switching distance. Its energy is then obtained by multiplying the *V*_*VdW*_ term with the scaling factor
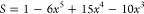

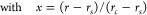
where *r*_*s*_ is the switching distance, and *r*_*c*_ is the cutoff distance.

Electrostatics without cutoff are implemented using the following potential
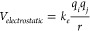
where  is Coulomb’s constant, *q*_*i*_ and *q*_*j*_ are the charges of the two atoms, and *r* is the distance between them. Electrostatics with cutoff are modified to use the reaction field method^30^ as follows
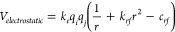

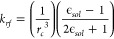

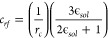
where *r*_*c*_ corresponds to the cutoff distance, and ϵ_*sol*_ corresponds to the solvent dielectric constant.

In addition to the above, TorchMD also trivially allows the use of any other external potential *V*_*ext*_ written in PyTorch which takes atomic coordinates as input and output energy and forces.

Thus, the total potential is calculated as
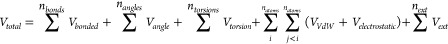
1

Since PyTorch offers automatic differentiation, there is no need to calculate analytical gradients from the forces. Forces can be obtained with a single autograd PyTorch call on the total energy of the system. Analytical gradients have been nevertheless implemented for all analytical AMBER potential terms for performance reasons.

### 2.3 Training Machine Learning Potentials

TorchMD provides a fully usable code for training neural network potentials in PyTorch called TorchMD-Net (github.com/torchmd/torchmd-net). Currently we are using a SchNet-based^4^ model. However, it would be straightforward to derive the forces from nonparametric kernel methods like FCHL,^34^ by providing a simple force calculator class, or other ML potentials. This object just takes as input the positions and box every time step and returns the external energies and forces computed with the method of choice.

For the present work, we took the featurization and atom-wise layer from SchNetPack^29^ but rewrote entirely the training and inference parts. In particular, to allow training on multiple GPUs, the network is trained using the PyTorch lightning framework.^35^ TorchMD can also run concurrently a set of identical simulations by just changing the random number generator seed, arranging the neural network potential into a batch for speed, thus recovering, at least partially, the efficiency of optimized molecular dynamics codes.

For the QM9 data set,^36^ we trained the model using a standard loss function using mean square error over the energies. For the coarse-grained model, training is performed using the bottom-up “force matching” approach, focused on reproducing thermodynamics of the system from atomistic simulations, as described in previous work.^8,9^

## 3 Results

To demonstrate the functionalities of TorchMD, here we present some application examples. First, a set of typical MD use cases (water box, small peptide, protein, and ligand) is used mainly to assess speed and energy conservation. Second, we validate the training procedure on QM9, a data set of 134k small molecule conformations with energies.^36^ In this case, however we cannot run any dynamical simulations as the data set only presents ground state conformations of the molecules, so we are mainly validating the training. Then, we demonstrate end-to-end differentiable capabilities of TorchMD by recovering force-field parameters from a short MD trajectory. Finally, we present a coarse-grained simulation of a miniprotein, chignolin,^37^ using NNP trained on all-atom MD simulation data. Here, we also describe how to produce a neural network-based coarse-grained model of chignolin, although the methods exposed are general to any other protein. A step-by-step example of the training and simulating CG model is presented in the tutorial available in the github.com/torchmd/torchmd-cg repository.

### 3.1 Simulations of All-Atom Systems and Performance

The performance of TorchMD is compared against ACEMD3,^31^ a high-performance molecular dynamics code. In Table 1, we can see the three different test systems comprised of a simple periodic water box of 97 water molecules, alanine dipeptide, and trypsin with the ligand benzamidine bound to it. As it can be seen, TorchMD is around 60 times slower on the test systems than ACEMD3 running on a TITAN V NVIDIA GPU. Most of the performance discrepancy can be attributed to the lack of neighbor lists for nonbonded interactions in TorchMD and is currently prohibitive for much larger systems as the pair distances cannot fit into GPU memory. This is not a strongly limiting factor for the CG simulations conducted in this paper as the number of beads remains relatively low for the test case. However, we believe that, with an upcoming implementation of neighbor lists, TorchMD can reach a much better performance, albeit still slower than highly specialized codes as ACEMD3 due to the generic nature of PyTorch operations in addition to the PyTorch library overhead.

**Table 1:** Performance Comparison for 50,000 Steps at 1 fs/timestep on Different Systems

system	atoms	TorchMD	ACEMD
water	291	6 min 56 s	7 s
alanine dipeptide	688	8 min 44 s	8 s
trypsin	3,248	13 min 2 s	16 s

Despite the low performance of the current TorchMD implementation, its end-to-end differentiability allows researchers to perform experiments which would not be possible with traditional much faster MD codes as demonstrated in the following sections.

To evaluate the correctness of the TorchMD implementation of the AMBER force field, we compared it against OpenMM for 14 different systems ranging from ions, water boxes, and small molecules to whole proteins, thus testing all the different force-field terms. In all 14 test cases, the potential energy difference between OpenMM and TorchMD was lower than 10^–3^ kcal/mol when computed with the same parameters. Energy conservation was validated with TorchMD by running an NVE simulation of a periodic water box for 1 ns with a 1 fs time step. Energy conservation normalized per degree of freedom was calculated as *E*_*total*_/*n*_*dof*_*R* where *n*_*dof*_ = 870 is the number of degrees of freedom of the system, and *R* is the ideal gas constant. We obtained a mean value of 1.1 × 10^–5^ K per degree of freedom showing a good energy conservation.

### 3.2 Training Validation on the QM9 Data Set

We trained and evaluated the performance on the QM9 benchmark data set^36^ in order to validate the training procedure of TorchMD-Net. QM9 comprises 133,885 small organic molecules with up to nine heavy atoms of type C, O, N, and F reporting several thermodynamic, energetic, and electronic properties for each molecule. We trained on the energy U0 and excluded 3,054 molecules due to failed geometric consistency checks as suggested by the data set. The remaining molecules were split into a training set with 110,000 samples and a validation set with 6,541 samples (5%), leaving 14,290 samples for testing.

**Figure 2:**
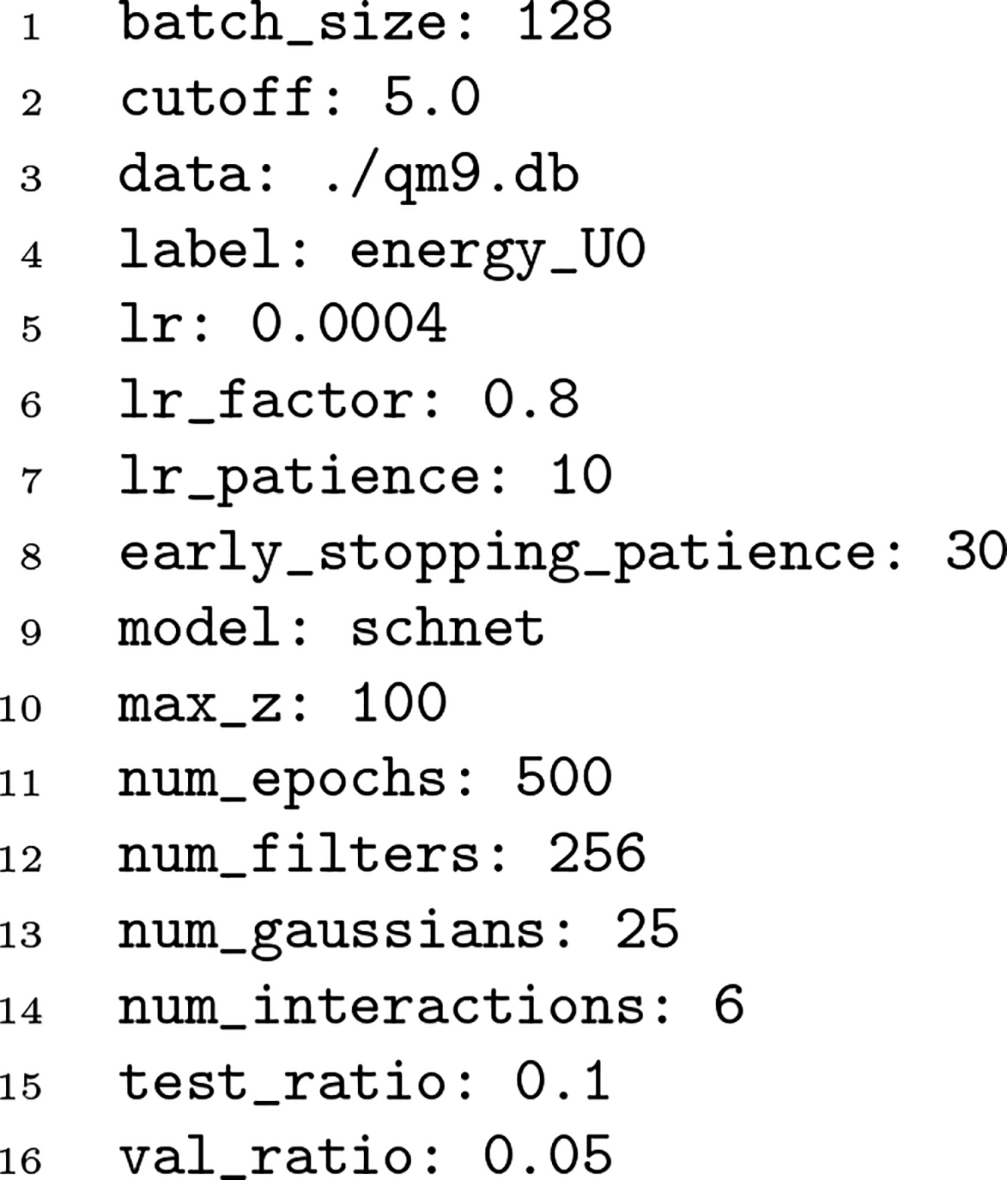
An example of a training input file for training QM9.

**Figure 3:**
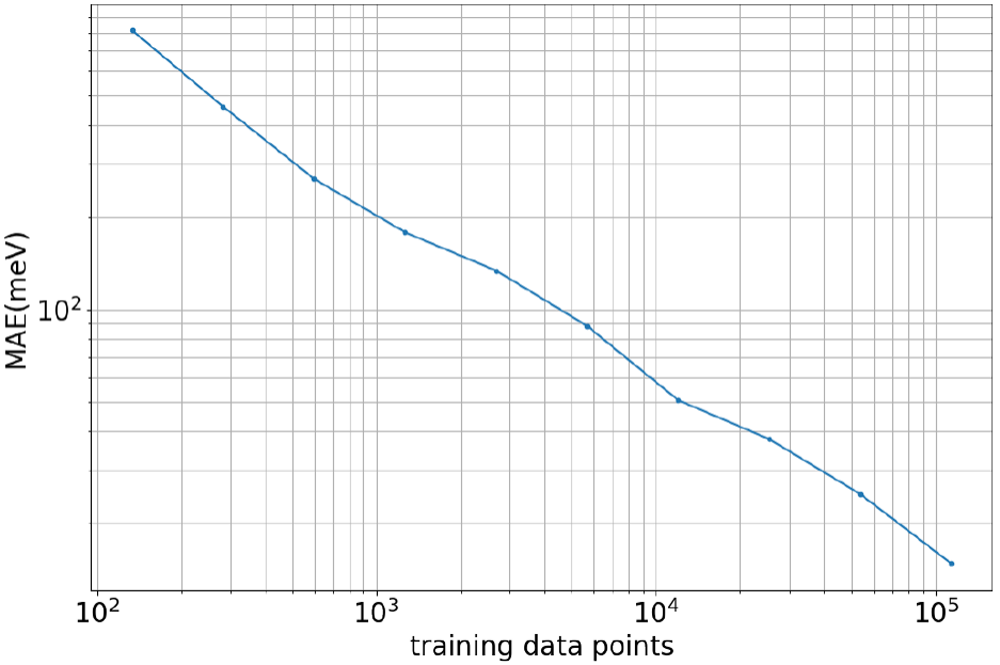
Learning curve for the QM9 data set.

The network was trained using an Adam optimizer^38^ with a learning rate scheduler on multiple GPUs by using PyTorch Lightning.^35^ An example of the configuration file for QM9 training is presented in Figure 2. We performed multiple trainings using TorchMD-Net with different amounts of training data (Figure 3). The learning rate scheduler was determined with a patience of 10 on a validation subset of 5% of all data chosen at random. The performance reported is for the randomly chosen test set. The linear shape of the test performance in the log–log scale demonstrates the correctness of the training.^39^ With the current set of hyperparameters (Figure 2), we report a best performance of 10 meV for 110,000 training points, marginally better than the reported best performance of SchNet for QM9.^29^

### 3.3 Demonstration of End-to-End Differentiable Simulations

The availability of automatic differentiation (AD) within a molecular dynamics package is beneficial beyond ML applications. Being able to compute gradients for all numerical operations opens up new avenues for sensitivity analysis, force-field optimization, and steered MD simulations, as well as simulations under highly complex constraints and restraints. To demonstrate these capabilities, the present example infers force-field parameters from a short MD trajectory.

First, a small water box containing 97 water molecules and one Na^+^/Cl^–^ ion pair was simulated using the TIP3P water model with flexible bonds and angles. After energy minimization and NVT equilibration at 300 K, the simulation was run for 10 ps in the microcanonical ensemble. The simulation used a 1 fs time step, a 9 Å cutoff with a 7.5 Å switch distance, and reaction field electrostatics. Coordinates and velocities were saved every 10 steps.

Next, all partial atomic charges *q* in the system were annihilated (in practice, they were scaled by 0.01 to ensure nonvanishing gradients of the electrostatic potential). In order to infer *q* from the MD trajectory, the integrator was initialized with snapshots *r*(*t*_*i*_), *v*(*t*_*i*_) from the trajectory. Then, 10 steps of simulation were run with the modified charges, and the final positions from this short simulation were compared with the respective subsequent trajectory snapshot *r*(*t*_*i*+1_). In other words, the simulation served as a parametrized propagator *Q*: (*r*(*t*), *v*(*t*); *q*)|→*r*(*t* + δ_*t*_) with δ_*t*_ = 10 fs. Due to the AD capabilities within TorchMD, this propagator is end-to-end differentiable.

**Figure 4:**
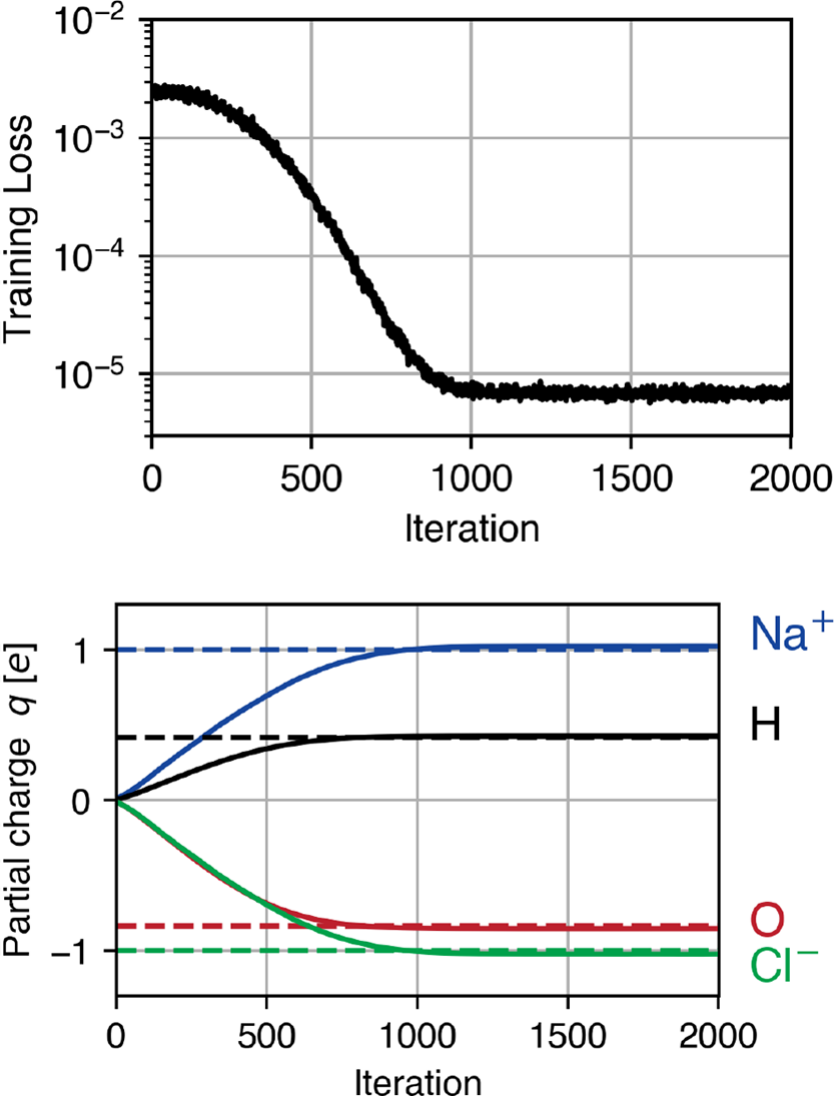
Inference of partial atomic charges *q* from a short trajectory. Training loss (top) and charges (bottom) during training.

To recover the charges, we minimized the loss function
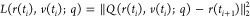
i.e., the mean-squared distance between the ground-truth trajectory and the propagated coordinates (taking into account periodic boundary conditions). This loss function is differentiable with respect to the charges *q* so that gradients can be obtained via backpropagation. Training was performed using Adam with a learning rate of 10^–3^ over one snapshot at a time. To enforce net neutrality, the positive charges (*q*_H_ and ) were implicitly obtained from the oxygen and chlorine charges, and only *q*_O_ and  were explicitly optimized. Figure 4 shows the evolution of the training loss and the partial atomic charges during training. After just one epoch (1000 iterations), the original charges were recovered up to 3% accuracy.

### 3.4 Coarse-Graining All-Atom Systems

**Figure 5:**
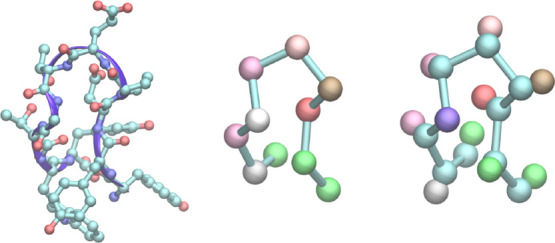
Miniprotein chignolin: heavy-atom representation (left) and coarse-grained representations: CA beads connected by bonds (middle) and CA and CB beads connected by bonds (right). The beads in coarse-grained representations were colored by bead type.

For our last application example, we built two coarse-grained models of chignolin: one solely based on α-carbon atoms (CA) and another one based on α-carbon and β-carbon atoms (CACB) (Figure 5).

#### 3.4.1 Training Data

We selected the CLN025 variant of chignolin (sequence YYDPETGTWY), which forms a β-hairpin turn while folded (Figure 5). Due to its small size (10 amino acids) and fast folding, it has been extensively studied with MD.^40−45^ Training data was obtained from an all-atom simulation of the protein in explicit solvent with ACEMD^31^ on the GPUGRID.net distributed computing network.^46^ The system containing one chignolin chain was solvated in a cubic box of 40 Å, containing 1881 water molecules and two Na^+^ ions. The system was simulated at 350 K with the CHARMM22* force field^47^ and the TIP3P model of water.^48^ A Langevin integrator was used with a damping constant of 0.1 ps^–1^. The integration time step was set to 4 fs, with heavy hydrogen atoms (scaled up to four times the hydrogen mass) and holonomic constrains on all hydrogen-heavy atom bond terms.^49^ Electrostatics were computed using Particle Mesh Ewald with a cutoff distance of 9 Å and a grid spacing of 1 Å. We used an adaptive sampling approach^50^ where new simulations were started from the least explored states. As a result, we obtained a total simulation time of 180 μs with forces and coordinates saved every 100 ps giving a total of 1.8 × 10^6^ frames.

To obtain the training data for the CA model, the initial training set of coordinates and forces was filtered to retain only CA atoms positions and forces. In this example, a coarse-grained system contains 10 beads, built out of seven unique types of beads, one for each amino acid type. The training set for the CACB model as prepared in a similar fashion, filtering both CA and CB atoms and achieving 19 beads and 8 unique types of beads, as all CA atoms was classified as one bead type with the exception of glycine, and each CB was assigned an amino acid-specific bead type. Details of bead selection for both models are described in Supporting Methods.

#### 3.4.2 Neural Network Potential Training

For coarse-grained simulations, it is important to provide some prior (fixed) potentials in order to limit the space that the dynamics can visit to the space sampled in the training data.^9^ All the terms of the force field could be applied, but for simplicity, we limit them to bonds and repulsions. Bonds prevent the protein polymer chain from breaking, and repulsions stop computing NNP on very close atom distances where there is no data.

For pairwise bonded terms, we used the all-atom training data to construct distance histograms for each pair of bonded bead types. Specifically, for each bonded pair, we counted the fraction of time that the respective distance spent in an equally spaced bin in a distance range appropriate to the bead selection, 3.55 and 4.2 Å for all bonds between α carbon beads and 1.3 and 1.8 Å for all bonds between α carbon and β carbon. The distance distributions were Boltzmann-inverted to obtain free-energy profiles, and these were used to fit the equilibrium distance *r*_0_ and the spring constant *k* of the respective harmonic potential
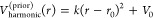
where *r* is the distance between the beads involved in the bond.

Prior potentials for nonbonded repulsive terms were derived analogously. Distance histograms were constructed with 30 equally spaced distance bins between 3 Å and 6 Å and were used to fit the parameter ϵ of the repulsive potential
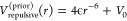
where *r*, as above, is the distance between the nonbonded beads. In fitting the potential curves, we corrected for the reference state by normalizing counts of each bin by the volume of the corresponding spherical shell. Nonlinear curve fits were performed with the Levenberg–Marquardt method of the SciPy package.^51^

The parameters of the prior forces are stored in a YAML force-field file. Plots presenting the quality of fits are included in the Supporting Information (Figures S1–S4) as well as YAML files describing the prior force field.

Based on the resulting prior force field and input coordinates, we calculated a set of prior forces acting on the beads and then deducted them from true forces, resulting in a set of forces that we refer to as delta-forces. Along with coordinates, delta-forces were used as the input for training. In the case of the CA model, embeddings correspond to integers unique for each amino acid type. For the CACB model, all α carbons have the same embedding with the exception of glycine, and each β carbon has an embedding unique for each amino acid type.

The network was trained using a force matching approach, where a predicted force is compared to a true force from the training set.^8,9^ In the example presented here, the network consisted of 3 interaction layers, 128 filters used in continuous-filter convolution, 128 features to describe atomic environments, a 9 Å cutoff radius, and 150 Gaussian functions for the CA model and 300 Gaussians for the CACB model as the basis set of the convolutions filters. Increasing the number of Gaussian functions for the CACB model was found to provide a higher stability of the model and prevent forming collapsed nonphysical structures during the simulation. Models for simulation were selected when the validation loss reached a plateau. The training and validation loss as well as learning rates are presented in Supporting Figure 5.

#### 3.4.3 Simulation of the NNP

**Figure 6:**
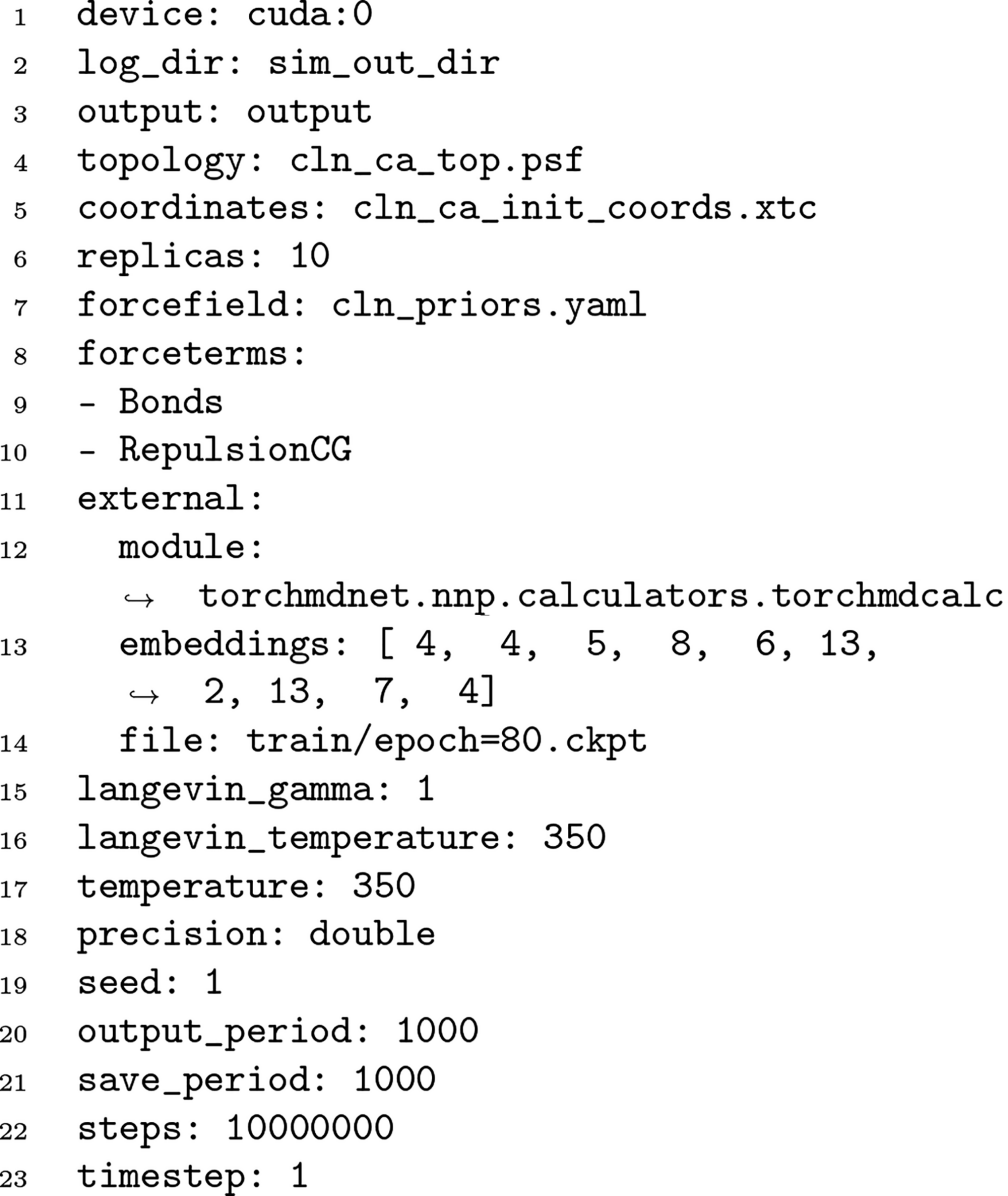
An example of a simulation input file.

The combinations of the force fields covering prior forces and the trained networks are used to simulate both CA and CACB systems with TorchMD. We introduce the parameters of the simulation as a YAML-formatted configuration file (Figure 6), although the simulation can be also started from the command line. The network is introduced to TorchMD as an external force, with the specified network’s location, embeddings, and a calculator. An external force calculator class must have a “calculate” method that returns a tuple with energy and forces tensors. In our case, for both models, we run the simulation at 350 K for 10 ns with a 1 fs time step, saving the output every 1000 fs. Note that while the simulations use a small time step, the effective dynamics of the coarse-grained systems is much faster than the all-atom MD system as the coarse-grained model is supposed to reproduce the energetics but with much faster kinetics. Since TorchMD can easily handle parallel dynamics, we concurrently run ten simulations, of which five start from the folded state and five start from unfolded conformations.

**Figure 7:**
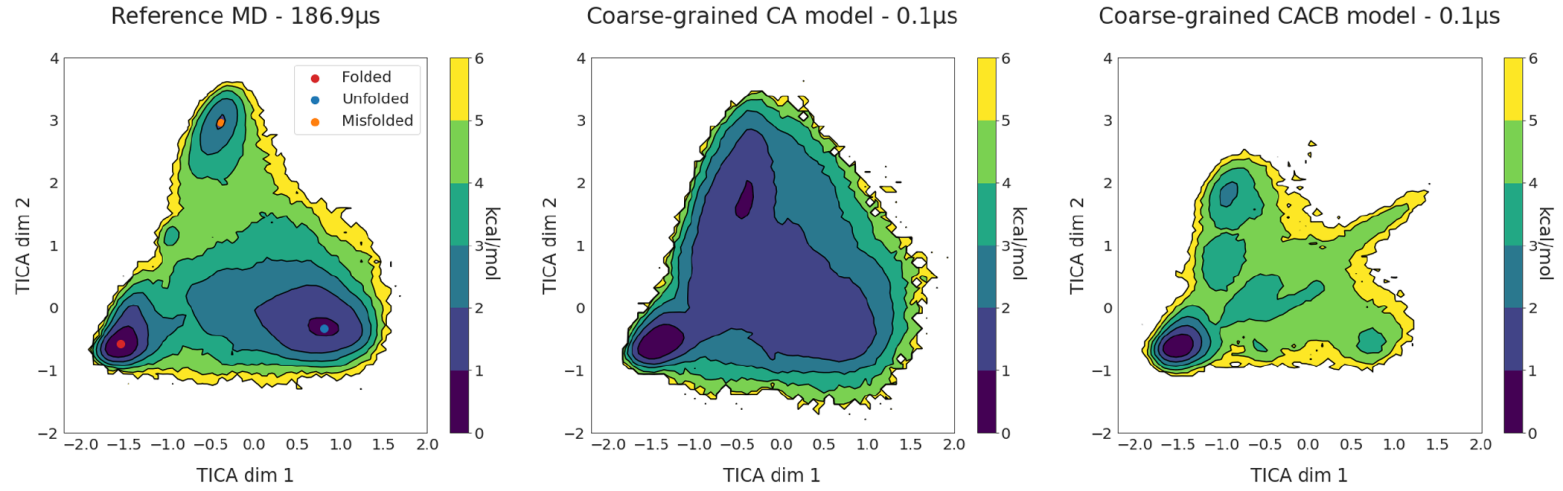
Two-dimensional free energy surfaces for the reference all-atom MD simulations (left) and the two coarse-grained models, CA (center) and CACB (right). The free energy surface for each simulation set was obtained by binning over the first two TICA dimensions, dividing them into a 120 × 120 grid, and averaging the weights of the equilibrium probability in each bin computed by the Markov state model. The reference MD simulations plot displays the locations of the three energy minima on the surface, corresponding to folded state (red dot), unfolded conformations (blue dot), and a misfolded state (orange dot). Both reference MD and coarse-grained simulations were performed at 350 K.

**Figure 8:**
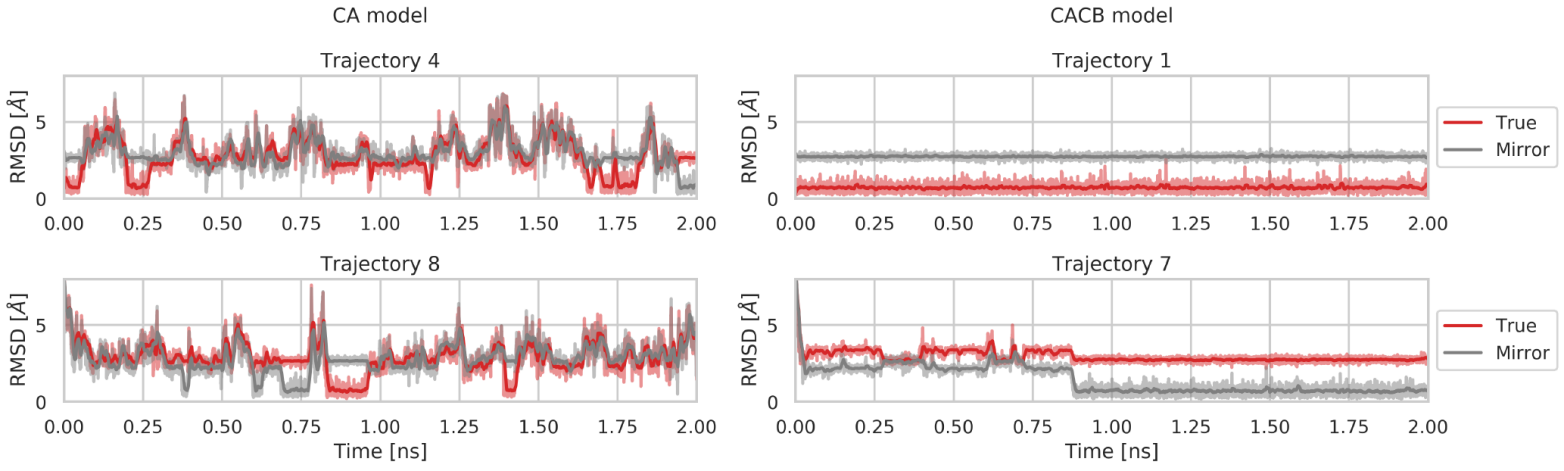
RMSD values across the first 2 ns of the unmodified trajectory (*True*, red) and a mirror image of the original trajectory (*Mirror*, gray) for the CA model (on the left) and the CACB model (on the right). Trajectory 4 (top left panel) and Trajectory 1 (top right panel) are examples of trajectories started from the folded state for the CA model and the CACB model, respectively. Trajectory 8 (bottom left panel) and Trajectory 7 (bottom right panel) are examples of trajectories started from the elongated chain for the CA model and the CACB model, respectively. A moving average of 100 frames is represented as darker lines. The full 10 ns of each simulation is included in Supporting Figures S6–S9.

The free energy surfaces obtained with a time-lagged independent component analysis (TICA)^52^ for the all-atom baseline simulations and the coarse-grained simulations obtained with TorchMD are presented in Figure 7. The energy landscapes are obtained from binning the configurations over the first two TICA dimensions and computing the average of the equilibrium probability on each bin, obtained by Markov state model analysis of the microstate of each configuration. To support TICA plots, we included plots with RMSD values for the first 2 ns of representative trajectories for both models with different starting points (Figure 8). Plots presenting full trajectories are included in the Supporting Information (Figures S6–S9). Neither SchNet nor prior energy terms can enforce chirality in the system, because they both work purely on the distances between the beads. Therefore, the RMSD plots were supplemented with RMSD values of the trajectory’s mirror image.

Results show that the coarse-grained simulations for both models were able to obtain several folding and unfolding events for chignolin. The energy landscapes for the CA model show that it captured the folded state as a global minimum of energy. The simulations also covers other minima representing unfolded and misfolded states. However, they do not recreate the energy barriers connecting these basins (as expected), which is better seen on the one-dimensional free energy surfaces (Figure S10). The CACB model also detects the global minimum correctly but fails at guessing the free energy of the unfolded region. Overall the simulation is less stable than for the CA model, and the misfolded state minimum is incorrectly located.

## 4 Conclusion

In this paper, we demonstrated TorchMD, a PyTorch-based molecular dynamics engine for biomolecular simulations with machine learning capabilities. We have shown several possible applications ranging from Amber all-atom simulations to end-to-end learning of parameters and finally a coarse-grained neural network potential for protein folding. In particular, building an NNP for protein folding requires supplementing it with asymptotic, analytical potentials for bonds and repulsions to prevent exploring conformations not visited in the training data in which the predictions of NNP are unreliable. We have shown how to coarse-grain a protein into either α-carbon atoms or α-carbon and β-carbon atoms. Currently, the CA model seems to work the best, but future research will indicate which models are better suited for a more diverse set of targets. TorchMD end-to-end differentiability of its parameters is a feature that projects such as the Open Force Field Initiative^53^ can potentially exploit. Furthermore, for additional speed, we plan to facilitate the integration of machine learning potentials in OpenMM^54^ and ACEMD^31^ and possibly develop a plug-in to extend support to more MD engines in the future. Meanwhile, we believe that TorchMD can play an important role by facilitating experimentation between ML and MD fields, speeding up the model-train-evaluate prototyping cycle, and promoting the adoption of data-based approaches in molecular simulations. All the code machinery to produce the models is made available for practitioners at github.com/torchmd.

## Supporting Information Available

Details of bead selection for both models (Supporting Methods), plots presenting quality of fits (Figures S1–S4), training and validation loss as well as learning rates (Figure S5), plots presenting full trajectories (Figures S6–S9), and one-dimensional free energy profile (Figure S10) (PDF)
