# Supplementary material for: TorchMD: A Deep Learning Framework for Molecular Simulations
Source: J Chem Theory Comput. 2021 Mar 17;17(4):2355–63. doi: 10.1021/acs.jctc.0c01343 (PMC8486166; doi:10.1021/acs.jctc.0c01343)
Supplement: Supplementary file 1 — ct0c01343_si_001.pdf [file ct0c01343_si_001.pdf]

Supplementary Information
TorchMD: A deep learning framework for molecular simulations

Stefan Doerr,[†] Maciej Majewski,[‡] Adrià Pérez,[‡] Andreas Krämer,[¶] Cecilia Clementi,[§,∥] Frank Noe,[¶,§,∥] Toni Giorgino,[⊥,#] and Gianni De Fabritiis[*,‡,†,@]

†*Acellera, Barcelona, Spain*

‡*Computational Science Laboratory, Universitat Pompeu Fabra, Barcelona, Spain*

¶*Department of Mathematics and Computer Science, Freie Universität, Berlin, Germany*

§*Department of Physics, Freie Universität, Berlin, Germany*

∥*Department of Chemistry, Rice University, Houston, Texas*

⊥*Biophysics Institute, National Research Council (CNR-IBF), Italy*

#*Department of Biosciences, Università degli Studi di Milano, Italy*

@*Institució Catalana de Recerca i Estudis Avançats, Barcelona, Spain*

E-mail: g.defabritiis@gmail.com

Supporting Methods

Coarse-Graining

Coarse-grained models were constructed based on only α-carbons (CA model) or both α- and β-carbons (CACB model) of chignolin. For CA model each α-carbon was assigned a bead_name based on the amino acid of origin, resulting in 7 unique bead types (Tab.S1). For CACB model all α-carbons, except for glycine, were classified as the same bead type and each β-carbon was assigned a bead_name based on the amino acid of origin, resulting in the total of 8 unique bead types (Tab.S2). These selections of beads were then used to filter the coordinates and forces from full-atomistic simulations and to compute parameters of the prior energy terms in the simulation force field. Each bead_name has an embedding associated with it that is later used as an input for the neural network.

Table S1: A set of coarse-grained beads building CA model, along with the embeddings required for the network.

index	atom_name	residue	bead_name	embedding
1	CA	TYR	CAY	4
2	CA	TYR	CAY	4
3	CA	ASP	CAD	5
4	CA	PRO	CAP	8
5	CA	GLU	CAE	6
6	CA	THR	CAT	13
7	CA	GLY	CAG	2
8	CA	THR	CAT	13
9	CA	TRP	CAW	7
10	CA	TYR	CAY	4

Table S2: A set of coarse-grained beads building CACB model, along with the embeddings required for the network.

index	atom_name	residue	bead_name	embedding
1	CA	TYR	CA	30
2	CB	TYR	CBY	4
3	CA	TYR	CA	30
4	CB	TYR	CBY	4
5	CA	ASP	CA	30
6	CB	ASP	CBD	5
7	CA	PRO	CA	30
8	CB	PRO	CBP	8
9	CA	GLU	CA	30
10	CB	GLU	CBE	6
11	CA	THR	CA	30
12	CB	THR	CBT	13
13	CA	GLY	CAG	31
14	CA	THR	CA	30
15	CB	THR	CBT	13
16	CA	TRP	CA	30
17	CB	TRP	CBW	7
18	CA	TYR	CA	30
19	CB	TYR	CBY	4

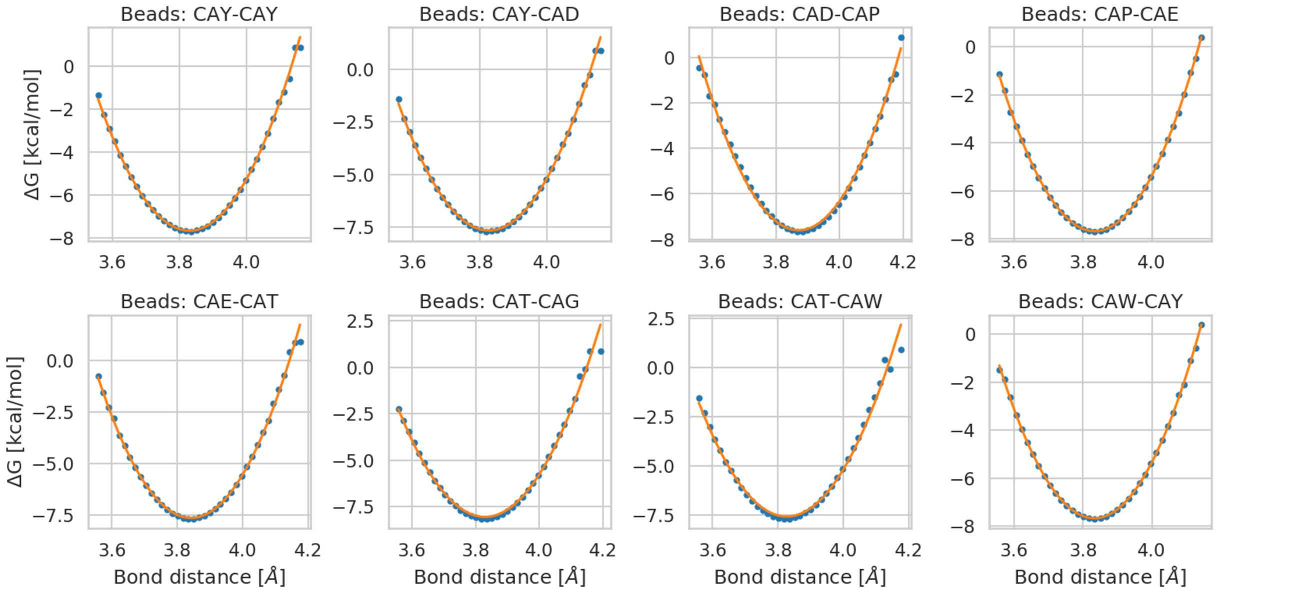

Figure S1: Each plot presents a fit of a function representing harmonic potential (orange line) to a free energy profile obtained form the distribution of distances of bonded α-carbon beads in full atom training data (blue dots). The fits were used to obtain force field parameters for bonded interactions in CA model.

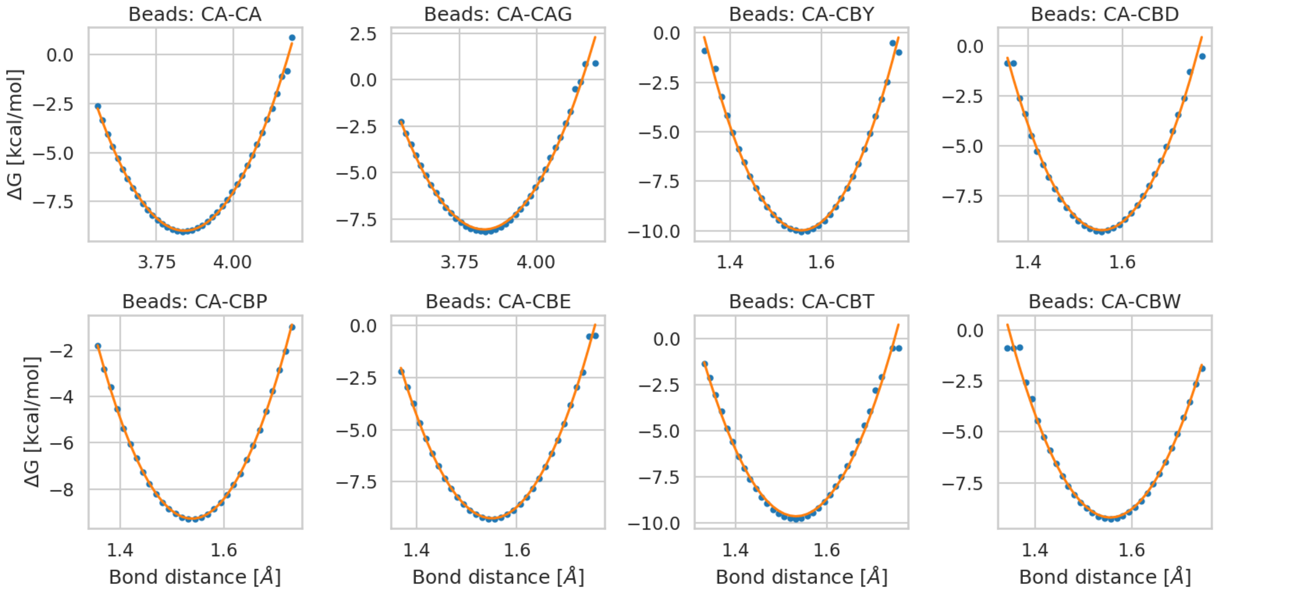

Figure S2: Each plot presents a fit of a function representing harmonic potential (orange line) to a free energy profile obtained form the distribution of distances of bonded α-carbon and β-carbon beads in full atom training data (blue dots). The fits were used to obtain force field parameters for bonded interactions in CACB model.

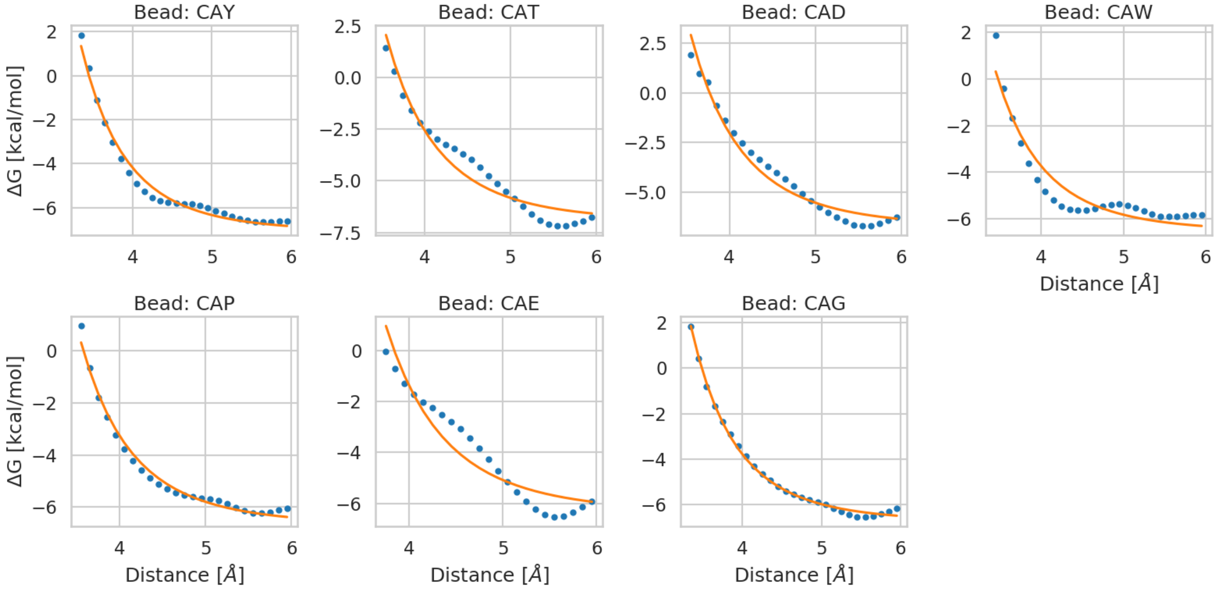

Figure S3: Each plot presents a fit of a function representing repulsive potential (orange line) to a free energy profile obtained form the distribution of distances between a given α-carbon atom and all non-bonded beads in full atom training data (blue dots). The fits were used to obtain force field parameters for non-bonded interactions in CA model.

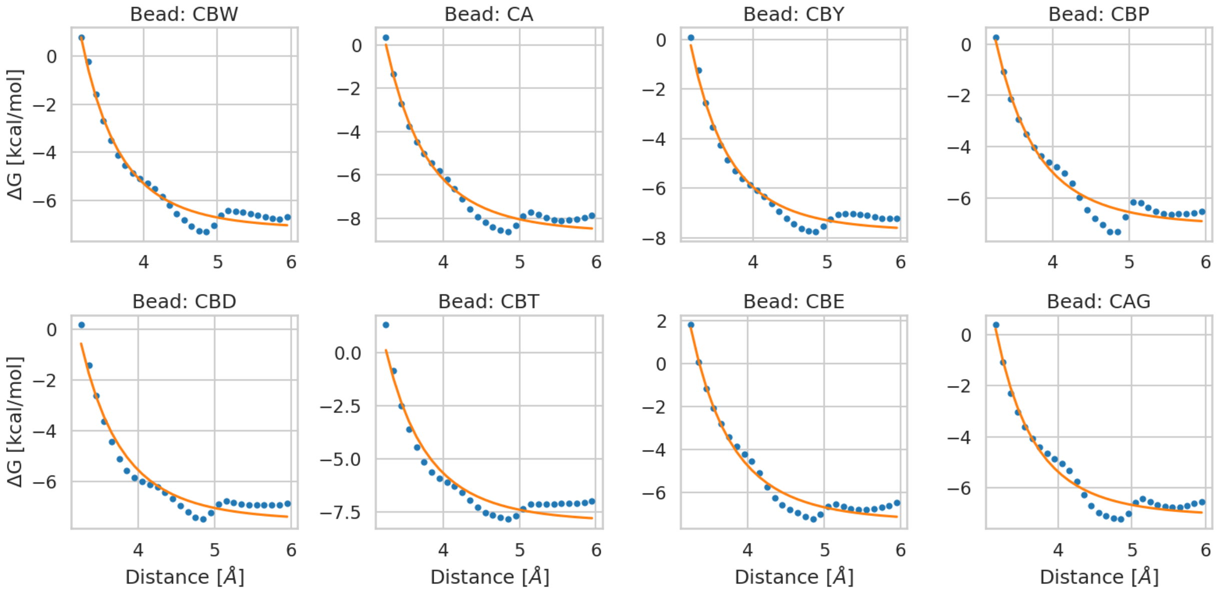

Figure S4: Each plot presents a fit of a function representing repulsive potential (orange line) to a free energy profile obtained form the distribution of distances between a given α-carbon or β-carbon atom and all non-bonded beads in full atom training data (blue dots). The fits were used to obtain force field parameters for non-bonded interactions in CACB model.

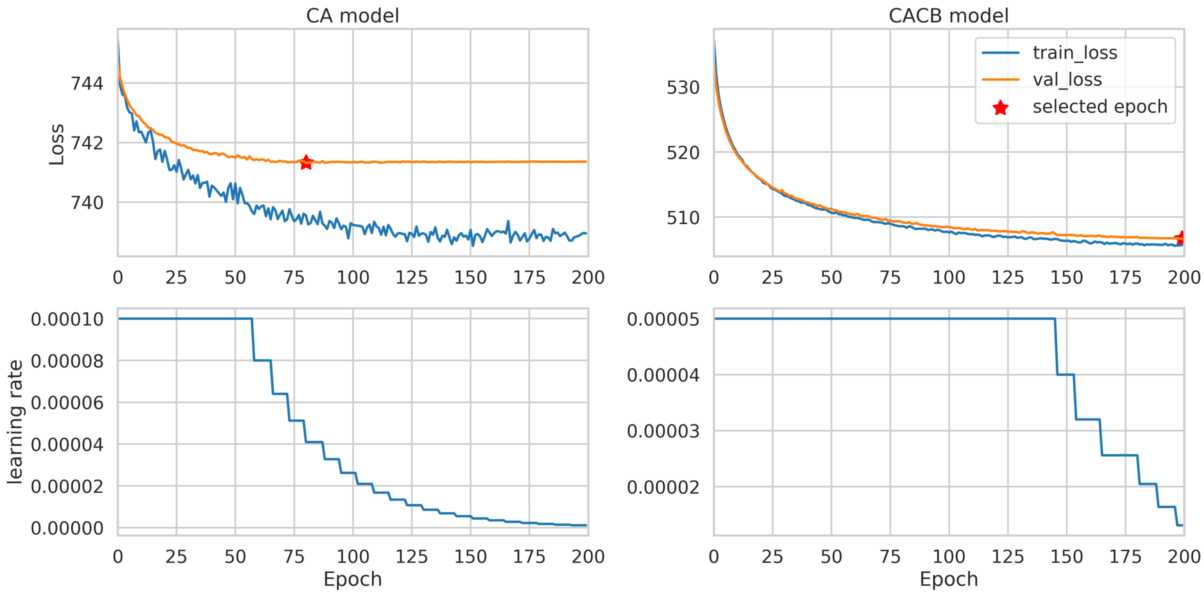

Figure S5: Top panel: Loss curves for CA model (left) and CACB model (right) of chignolin. Blue curves represent training loss values (*train_ loss*) and orange curves represent validation loss values (*val_ loss*). The models selected are marked with a red star. Bottom panel: learning rate values across the training of the corresponding models.

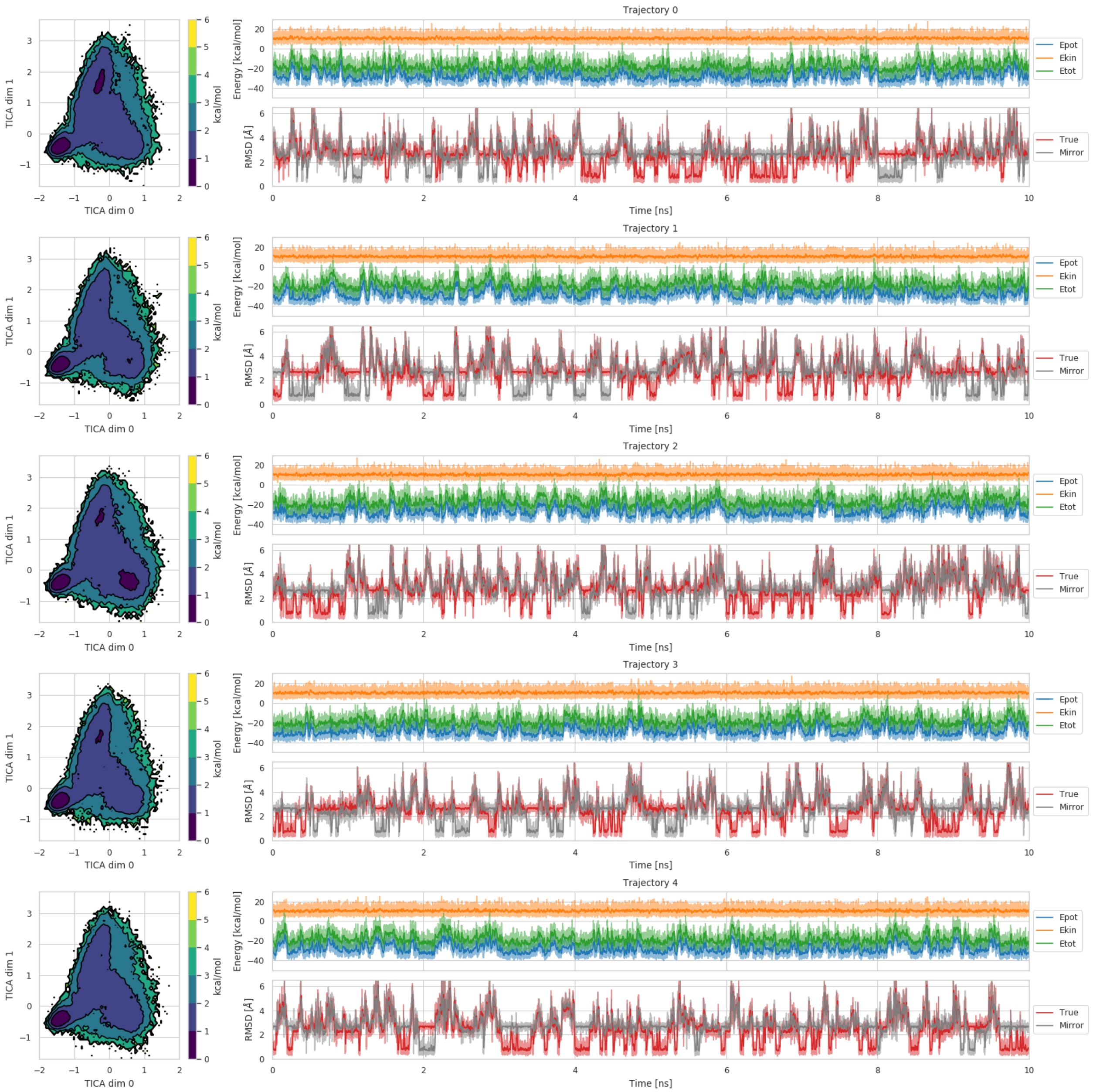

Figure S6: Full trajectories simulated with neural network for CA model starting from folded conformation. Each one of five panels represents a full analysis of energy (top right plot) and RMSD (bottom right plot) across the 10 ns simulation. The energy plot presents potential energy (*Epot*, blue), kinetic energy (*Ekin*, orange) and total energy (*Etot*, green). The RMSD plot presents RMSD values across the simulation for the unmodified trajectory (*True*, red) and a mirror image of the original trajectory (*Mirror*, gray). A moving average of 100 frames is represented as darker lines. The left plot on each panel presents a two-dimensional free energy surfaces for the trajectory.

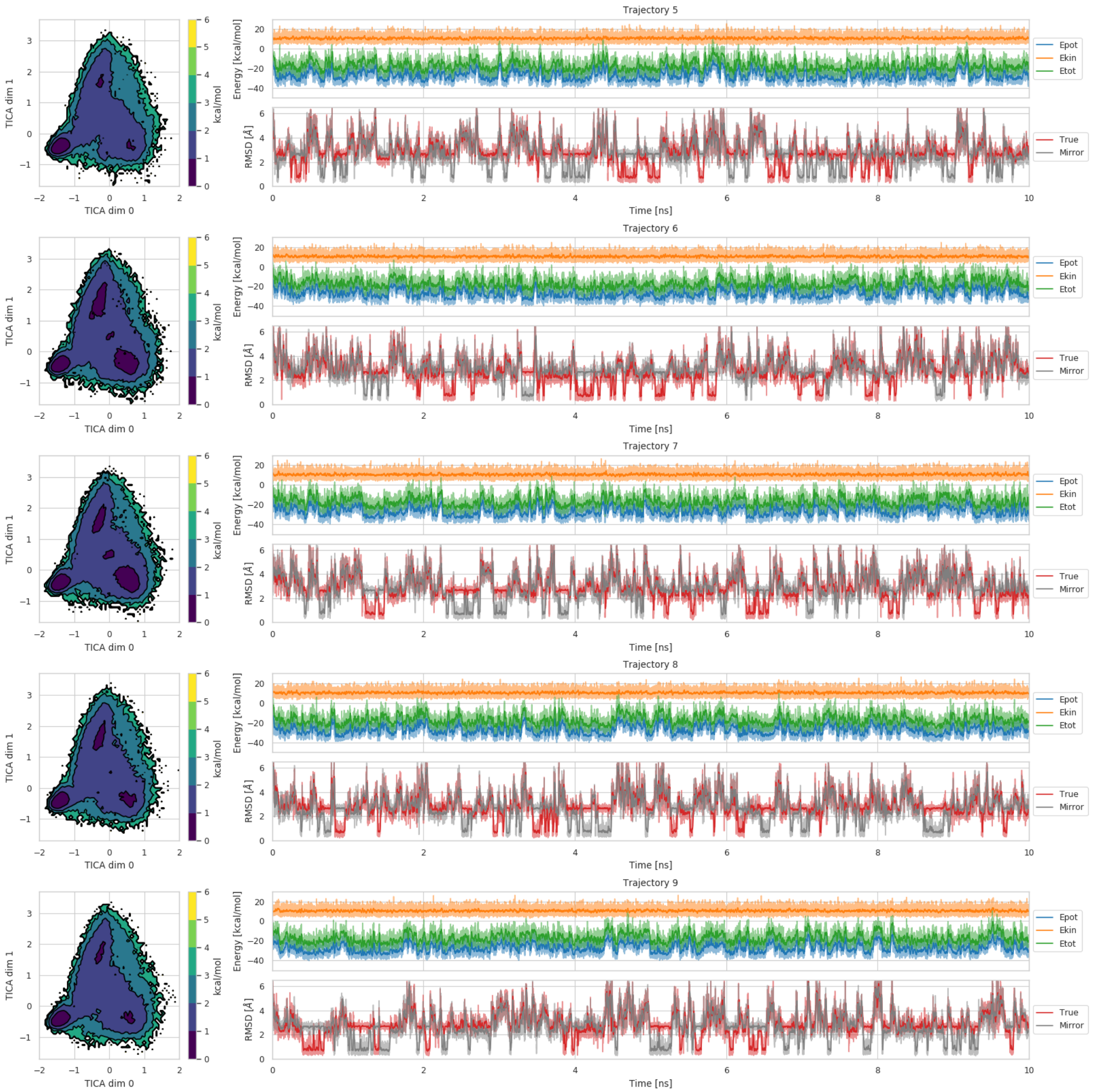

Figure S7: Full trajectories simulated with neural network for CA model starting from an elongated chain. Each one of five panels represents a full analysis of energy (top right plot) and RMSD (bottom right plot) across the 10 ns simulation. The energy plot presents potential energy (*Epot*, blue), kinetic energy (*Ekin*, orange) and total energy (*Etot*, green). The RMSD plot presents RMSD values across the simulation for the unmodified trajectory (*True*, red) and a mirror image of the original trajectory (*Mirror*, gray). A moving average of 100 frames is represented as darker lines. The left plot on each panel presents a two-dimensional free energy surfaces for the trajectory.

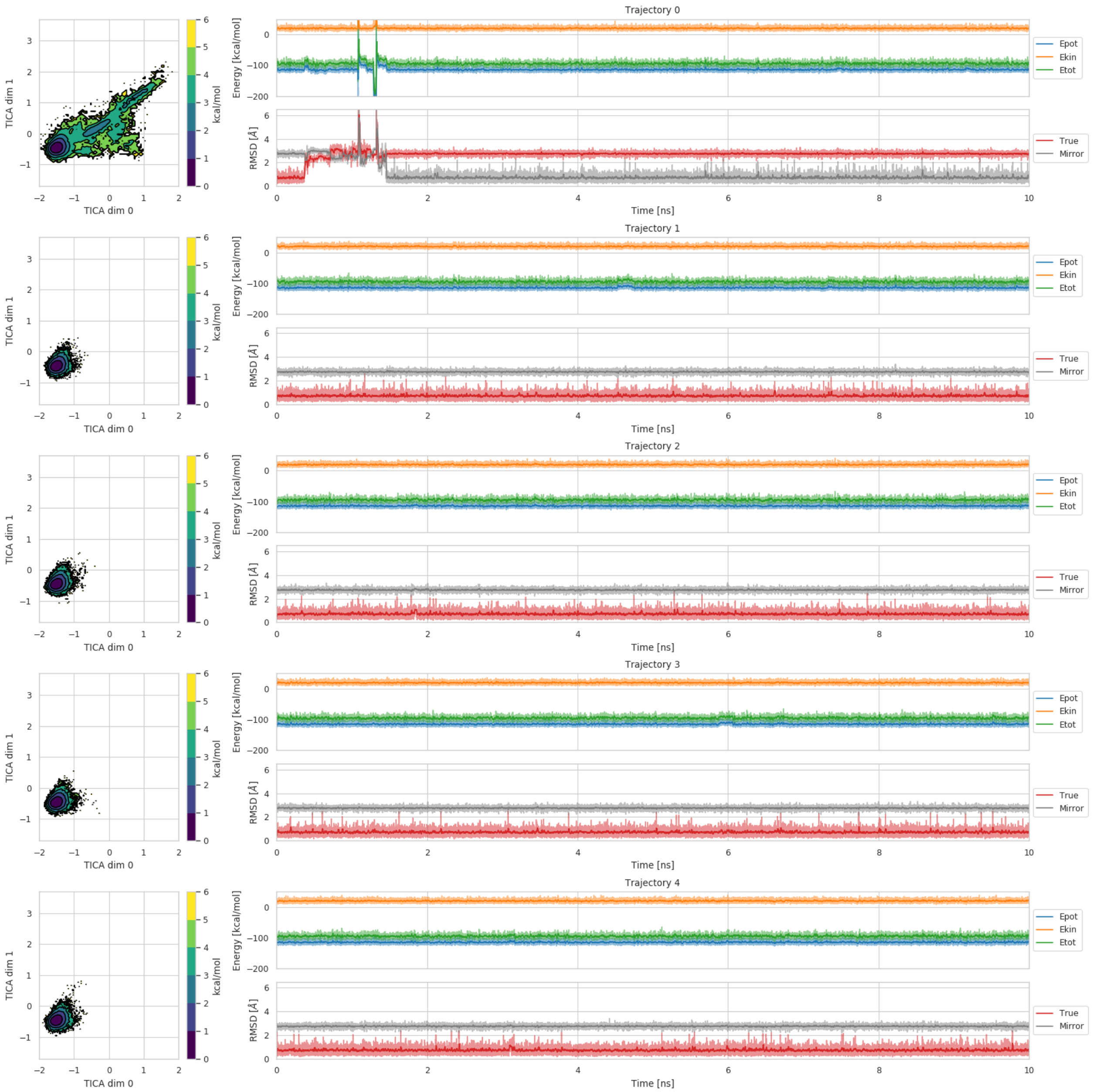

Figure S8: Full trajectories simulated with neural network for CACB model starting from folded conformation. Each one of five panels represents a full analysis of energy (top right plot) and RMSD (bottom right plot) across the 10 ns simulation. The energy plot presents potential energy (*Epot*, blue), kinetic energy (*Ekin*, orange) and total energy (*Etot*, green). The RMSD plot presents RMSD values across the simulation for the unmodified trajectory (*True*, red) and a mirror image of the original trajectory (*Mirror*, gray). A moving average of 100 frames is represented as darker lines. The left plot on each panel presents a two-dimensional free energy surfaces for the trajectory.

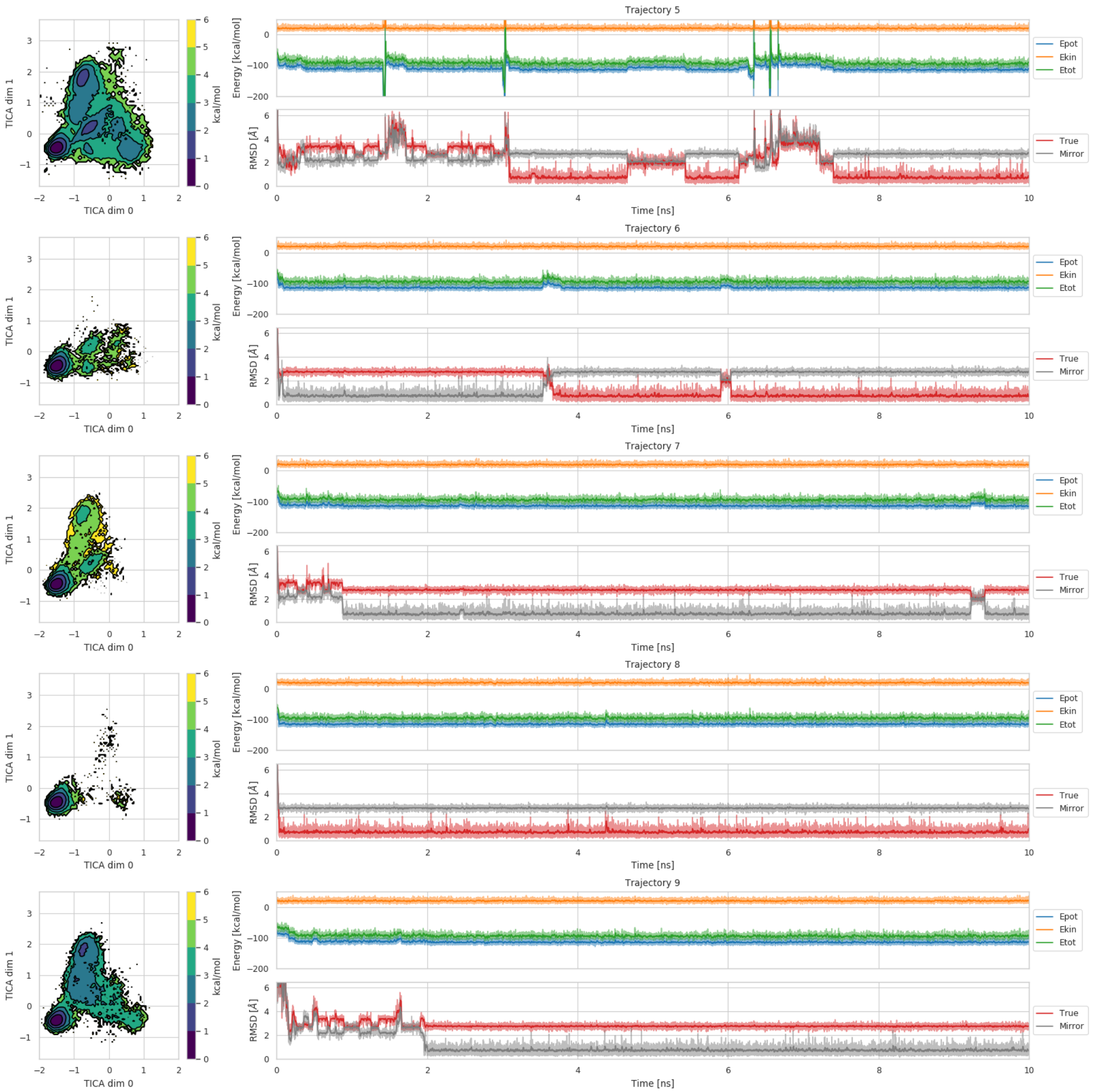

Figure S9: Full trajectories simulated with neural network for CACB model starting from an elongated chain. Each one of five panels represents a full analysis of energy (top right plot) and RMSD (bottom right plot) across the 10 ns simulation. The energy plot presents potential energy (*Epot*, blue), kinetic energy (*Ekin*, orange) and total energy (*Etot*, green). The RMSD plot presents RMSD values across the simulation for the unmodified trajectory (*True*, red) and a mirror image of the original trajectory (*Mirror*, gray). A moving average of 100 frames is represented as darker lines. The left plot on each panel presents a two-dimensional free energy surfaces for the trajectory.

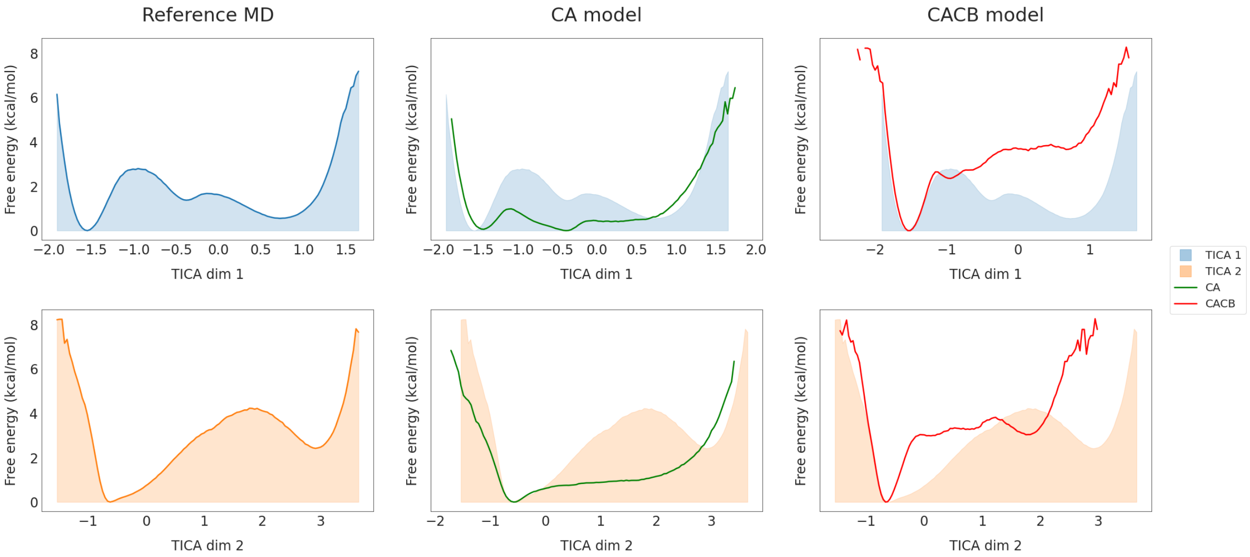

Figure S10: One-dimensional free energy surfaces of the first two TICA dimensions for the reference MD simulations (left), CA model (center) and CACB model (right). First row corresponds to the first TICA dimension while the second row corresponds to the second TICA dimension. The plot was performed the same way as the two-dimensional one (Fig.6), but only binning a single dimension. Coarse-grained models surface lines are depicted with a specific color (green for CA model, red for CACB model). Surfaces for the reference MD simulations are displayed at each plot as a shade for comparison with the coarse-grained surfaces.
